# Impact of Genetic Polymorphism of *methylenetetrahydrofolate reductase C677T *on Development of Hyperhomocysteinemia and Related Oxidative Changes in Egyptian β-Thalassemia Major Patients

**DOI:** 10.1371/journal.pone.0155070

**Published:** 2016-05-17

**Authors:** Mai A. Abd-Elmawla, Sherine M. Rizk, Ilham Youssry, Amira A. Shaheen

**Affiliations:** 1 Department of Biochemistry, Faculty of Pharmacy, Cairo University, Cairo, Egypt; 2 Department of Pediatric Hematology, Faculty of Medicine, Cairo University, Cairo, Egypt; Kermanshah University of Medical Sciences, ISLAMIC REPUBLIC OF IRAN

## Abstract

**Background:**

β-thalasemia major (β-TM) patients often suffer from various vascular complications together with increased oxidative stress. Hyperhomocysteinemia (Hhcy) has been defined as a risk factor for these complications. Genetic polymorphism of methylenetetrahydrofolate reductase *(MTHFR) C677T* has been shown to cause Hhcy particularly in individuals with low B-vitamins. However, the status of homocysteine (hcy) in β-TM has not yet been adequately defined.

**Aim:**

To evaluate the genetic polymorphism of *MTHFR C677T* among β-TM patients and its prospective contribution to Hhcy and related oxidative changes.

**Subjects and Methods:**

Genotyping for *MTHFR C677T* was done by PCR-RFLP technique. Plasma hcy, vitamin B12, folate, malondialdehyde (MDA), total antioxidant capacity (TAC), oxidized low density lipoprotein (oxLDL), total nitric oxide (NOx) and lipid profile were determined in 66 β-TM patients and 66 control subjects of matched age and sex.

**Results:**

The prevalence of *MTHFR 677TT* genotype was significant among β-TM patients (12%) compared to (3%) controls (OR = 4.9, 95%CI:1.2–24.2,P = 0.03). A strong association between Hhcy and *MTHFR TT* genotype was observed (OR = 7.7, 95%CI:2.8–20.9) where all β-TM patients with *TT* genotype were hyperhomocystienemic (≥ 15 μmol/l) and having sub-optimal folate level than those with *CT* or *CC* genotypes. Hyperhomocystienemic patients have suffered from increased oxidative stress characterized by significant increase in plasma MDA and oxLDL, and a significant reduction of plasma TAC and total NOx. Lipid profile of those patients was severely affected indicated by reduction in HDL and HDL/LDL and elevation in atherogenic index as compared with *CC* genotype. Other measured parameters were not significantly different among β-TM patients with different *MTHFR* genotypes.

**Conclusion:**

This study suggests that Egyptian β-TM patients with *MTHFR 677TT* genotype could be at increasing risk of developing Hhcy particularly with folate deficiency. This state of Hhcy may account potentially for most oxidative changes and atherogenic vascular complications frequently reported in β-TM patients.

## Introduction

β-thalassemia is an inherited disorder caused by mutations in the β-globin genes leading to a total lack or reduction in the synthesis of normal β-globin chains. β-thalassemia is a major public health problem in Egypt, where the carrier rate was 9%-10% [1]. The genetic disorder includes a wide variety of clinical phenotypes, ranging in severity from clinically silent heterozygous β-thalassemia to severe transfusion-dependent β-thalassemia major (β-TM) [2]. Patients with β-TM present clinically with severe transfusion-dependent anemia together with other related complications including endothelial and vascular dysfunctions, increased oxidative stress with subsequent atherosclerotic development and cardiovascular diseases [3,4]. Elevated plasma level of homocysteine (hcy) has been defined as an independent risk factor for development of these complications [5].

Several polymorphisms of genes encoding for enzymes acting in the remethylation pathway of hcy metabolism such as *methylenetetrahydrofolate reductase (MTHFR) C677T* have been shown to cause hyperhomocysteinemia (Hhcy) particularly in patients with deficiency of folate, vitamin B6 and B12 [6]. *MTHFR* affects the NADPH-linked reduction of *5*,*10-methylene tetrahydrofolate* to *5-methyltetrahydrofolate*. A *C* to *T* missense mutation at nucleotide *677* produces a thermolabile form of the enzyme associated with reduction in its activity due to alanine to valine substitution [7]. This can lead to elevated hcy which acts as prooxidant, generates free radicals by auto-oxidation, induces lipid peroxidation [8], decreases endothelial NO and causes endothelial cell damage [9]. Hhcy has been claimed to have a part in the aetiopathogensis of several disorders including most importantly cardiovascular and peripheral vascular diseases [10].

Oxidative stress, defined as an imbalance in equilibrium between pro- and antioxidant system, is important in the pathology of many diseases [11]. Thalassemia is accompanied by increased oxidative stress where the increment in serum ferritin due to continuous blood transfusions promotes peroxidative damage in thalassemia patients [12]. Ferritin can also catabolize folate *in vitro* and *in vivo* [13]. Furthermore, in the presence of iron, hcy exhibits more significant prooxidative activity and causes gradually oxidative modification of low density lipoprotein (LDL) with increased formation of thiobarbituric acid reactive substances [12]. All these factors are likely to trigger a cascade of events leading to various atherogenic vascular complications often reported in β-TM (3).

The status of hcy in patients with thalassemia has not yet been adequately defined. Some studies showed no change in plasma hcy [4,14] and others showed decreased level [12] compared to healthy control subjects. The prevalence of *MTHFR C677T* gene mutation which is closely linked to Hhcy has been reported in other pathological disorders (7), however their association with β-TM is still debated. Therefore the current study conducted to evaluate the prevalence of genetic polymorphism of *MTHFR C677T* among Egyptian β-TM patients and its prospective contribution to Hhcy as well as the related oxidative changes in these patients.

## Subjects and Methods

### Study Subjects

Sixty six patients (30 males, 36 females) with β-TM aged 8 to 26 years with a mean age (15.4 ± 0.69 years) were recruited consecutively from the Pediatric Hematology Clinic at Cairo University, Egypt between the periods of August 2012 to May 2013. All patients were clinically diagnosed based on onset of disease, hemoglobin (Hb) level prior to blood transfusion and Hb electrophoresis. Each patient in the study had a clinical and hematological data file including diagnosis, duration of disease, body weight, height, family and medication history, liver and spleen status. Data of serum ferritin, creatinine, liver function tests, and complete blood count were all extracted. All patients received blood transfusion every 4 weeks, standard iron-chelation therapy (deferoxamine), calcium and folic acid supplements. A total of 42 out of 66 of β-TM patients (64%) were splenectomized, 30 patients (45%) had mongoloid features, and 20 patients (30%) were with hepatomegaly. A control group of 66 healthy volunteers (34 males, 32 females) without any histories of chronic inflammatory diseases or hematologic disorders aged 9 to 28 years with a mean age (16.3 ± 1.2 years) were randomly selected from healthy blood donors and school students who came for routine checkups.

### Ethics

The study was approved by the Research Ethics Committee for experimental and clinical studies at Faculty of Pharmacy, Cairo University, Cairo, Egypt. Patients and control subjects above 18 years old were asked to give written informed voluntary consent to participate in the study. In those below 18 years of age, written informed consent was obtained from the parents according to the protocol approved by the local ethics committee and in accordance with the ethical standard laid down in the Helsinki declaration.

### Sampling

All participants in this study didn't receive blood transfusion within at least one month prior to the blood collection. Venous blood samples were collected from all participants in EDTA tubes after an overnight fasting. An aliquot of whole blood was used for DNA extraction and the other blood was rapidly centrifuged at 3000 rpm for 10 min in cold centrifuge, plasma was then separated immediately and stored at -80°C until further analysis.

### Biochemical Analysis

Total plasma hcy was determined by competitive ELISA kit (IBL International, Germany) as described by Frantzen et al. [15]. Plasma Vitamin B12 was estimated by a double–antibody sandwich ELISA kit (Glory Science, USA), whereas plasma folic acid was estimated using competitive ELISA kit (WKEA MED, USA). Total cholesterol (TC) was measured using established enzymatic methods using StanBio kit (USA) [16]. High density lipoprotein-cholesterol (HDL-C) was estimated by HDL-C precipitant method [17]. Triglyceride (TG) was assessed enzymatically [18]. LDL-C concentrations were determined by Friedewald's formula [19]. Atherogenic index of plasma (AIP) was calculated as log (TG/HDL-C) [20]. Plasma oxidized low density lipoprotein (oxLDL) was estimated by sandwich ELISA kit (Immundiagnostik, Germany). Plasma total antioxidant capacity (TAC) was estimated by colorimetric kit (Immundiagnostik, Germany). Plasma malondialdehyde (MDA) was estimated as index of lipid peroxidation [21]. Plasma nitrate/nitrite (NOx) as index of nitric oxide was estimated by Griess reaction using commercial kit (R&D Systems, USA) as described by Miles et al. [22].

### Genotype Analysis of *MTHFR C677T* using PCR-RFLP

**Fig 1:**
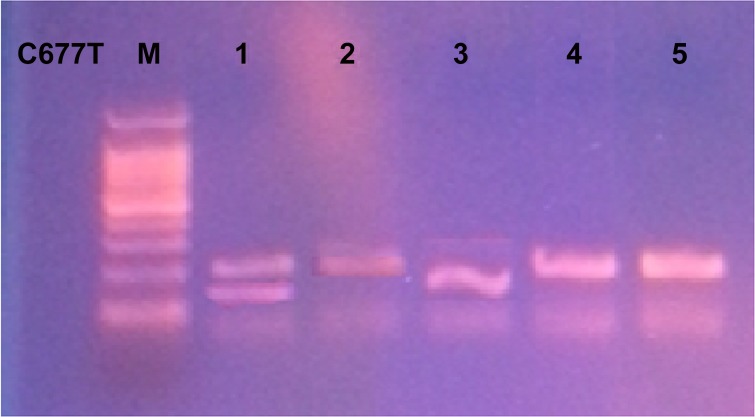
Detection of *MTHFR C677T* gene polymorphism. M: DNA molecular weight marker. Lane 5: PCR product before Hinf I RFLP at 198bp. Lanes 4&2: Wild type *CC*. Lane1: Heterozygous (*CT*). Lane 3: Homozygous (*TT*). RFLP Restriction Fragment Length Polymorphism

DNA was extracted from whole blood using DNA extraction Kit (Qiagen, Valencia, CA). Genotyping was performed according to previously described method [23]. DNA was amplified with the forward primer *5’-TGAAGGAGAAGGTGTCTGCGGGA-3*’ and the reverse primer *5’-AGGACGGTGCGGTGAGAGTG-3’*. PCR thermal cycling conditions were 5 min denaturation at 94°C then 40 cycles of 94°C for 30sec, 62°C for 30sec, and 72°C for 30sec followed by 7min extension at 72°C. Amplified 198-bp PCR products were digested with *Hinf I* (Invitrogen, USA) and visualized under electrophoresis on a 2% of agarose gel with ethidium bromide. The *C* allele produced a 198-bp band, and the *T* allele produced 175- and 23-bp fragments (Fig 1).

### Statistical Analysis

Data were analyzed using SPSS software version 17. The results were expressed as mean ± SEM and statistical comparisons were carried out using unpaired Student’s *t*-test and one way analysis of variance test (ANOVA) followed by Tukey's HSD test. Using logistic regression analysis, odds ratio (OR) were calculated with 95% confidence interval (CI). Scatter plots were used to show the levels of hcy and micronutrients among different groups, where the limit for folate deficiency was set at <11 μg/l [24] and for vitamin B12 was set at < 200 ng/l [25]. The associations between variables were assessed by Pearson’s correlation coefficient (2- tailed). The level of significance was identified at P<0.05.

## Results

### Demographic and Biochemical Measurements of Patients with β-TM and Controls

As expected in β-TM patients, Hb, packed cell volume and RBCs count were significantly lower, while platelets and WBCs counts were significantly higher than the control subjects. The plasma transaminases (AST and ALT) activities were significantly increased as well as serum ferritin level was dramatically elevated among patients with β-TM compared to healthy controls (Table 1).

**Table 1:** Demographic characteristics, hematological, and biochemical parameters of β-TM patients and controls.

	β-TM patients (n = 66)	Controls (n = 66)	P values
**Age (years)**	15.40 ± 0.69	16.30± 1.20	0.43
**BSA (m**^**2**^**)**	1.22 ± 0.03	1.36 ± 0.04	0.85
**Hb (g/dl)**	6.90 ± 0.12	14.05 ± 0.25	0.04*
**PCV (%)**	24.10 ± 0.44	41.60 ± 0.39	0.001**
**RBCs Count(10**^**6**^**/Cmm)**	3.05 ± 0.07	4.90 ± 0.09	0.03*
**Platelets (×10**^**9**^**/L)**	602 ± 29.50	239 ± 15.50	<0.0001**
**WBCs (×10**^**9**^**/L)**	13.60 ± 0.68	8.60 ± 0.19	<0.0001**
**ALT (IU/l)**	65.10 ± 6.20	22.90 ± 1.70	<0.0001**
**AST (IU/l)**	56.70 ± 4.50	24 ± 1.50	<0.0001**
**ALP (IU/l)**	66.80 ± 6.60	57.80± 5.10	0.09
**Total bilirubin (mg/dL)**	2.02 ± 0.10	0.70 ± 0.03	<0.0001**
**Creatinine (mg/dL)**	1.20 ± 0.18	0.80 ± 0.03	<0.0001**
**Ferritin (**μ**g/l)**	1854 ± 130	52.40 ± 3	<0.0001**

Data are represented as mean ± SE. Statistically significant at

* P<0.05

** P< 0.01. ALT alanine transaminase, ALP alkaline phosphatase, AST aspartate transaminase, BSA body surface area, β-TM thalassemia major, Hb hemoglobin, PCV packed cell volume, RBCs red blood cells, WBCs white blood cells.

Plasma hcy level was significantly higher (p = 0.004) among patients with β-TM together with a significant reduction of plasma vitamin B12 (p<0.0001) and folic acid (p = 0.03) compared to control group. Regarding lipid profile, plasma TC and TG levels were not significantly changed in patients with β-TM from those of the control subjects. In spite of these results, patients with β-TM had higher risk for atherosclerosis than normal subjects, where LDL-C (p<0.0001) and the AIP (p<0.0001) were significantly increased, while HDL-C (p<0.0001) and HDL/LDL (p<0.0001) were significantly decreased in patients with β-TM compared to controls. Data also revealed that patients with β-TM were in a state of oxidative stress characterized by marked elevation of plasma oxLDL (P = 0.01) and MDA (P<0.0001) together with a significant reduction in plasma TAC (P = 0.03) and total NOx (P<0.0001) compared with the control values (Table 2).

**Table 2:** Biochemical parameters of β-TM patients and controls.

	β-TM patients (n = 66)	Controls (n = 66)	P values
**hcy (**μ**mol/l)**	15.38±1.07	10.07±0.71	0.004**
**Vitamin B12 (ng/l)**	233.60 ±18	395.60 ±29	<0.0001**
**Folate (**μ**g/l)**	5.38± 0.63	8.17±1.24	0.03*
**TC (mg/dl)**	195.40±7.40	173±8.10	0.2
**TG (mg/dl)**	115±2.50	109±4.40	0.5
**HDL-C (mg/dl)**	42.60±1.70	75.85±4.70	<0.0001**
**LDL-C (mg/dl)**	125.10 ± 7.40	75.60 ± 6.30	<0.0001**
**AIP**	0.43 ± 0.01	0.20 ± 0.03	<0.0001**
**HDL/LDL**	0.47 ± 0.06	1.14 ± 0.12	<0.0001**
**oxLDL (ng/ml)**	219.40±14.70	155.90±19.20	0.01*
**MDA (nmol/ml)**	16.20±0.54	9.37±1.40	<0.0001**
**TAC (**μ**mol/l)**	291.60 ± 8	325.90 ±10	0.03*
**Total NOx (**μ**mol/l)**	133 ± 8.60	186±10.20	<0.0001**

Data are represented as mean ± SE. Statistically significant at

* P<0.05

** P< 0.01. AIP Atherogenic index of plasma, β-TM thalassemia major, hcy homocysteine, HDL-C high density lipoprotein-cholesterol, LDL-C low density lipoprotein-cholesterol, MDA malondialdehyde, oxLDL oxidized low density lipoprotein, TAC total antioxidant capacity, TC total cholesterol, TG triglycerides, total NOx total nitric oxide.

### Prevalence of *MTHFR C677T* among β-TM Patients and Controls

The frequency of the homozygous variant genotype *MTHFR 677TT* was higher in patients with β-TM (12%) than controls (3%) with an OR of 4.9 (95%CI:1.2–24.2). The allelic frequency for *MTHFR T* variant allele was higher (20%) in patients with β-TM than controls (7%) with an OR of 3.3 (95% CI of 1.5–7.4) (Table 3).

**Table 3:** Genotype and allele frequency distribution of *MTHFR C677T* in β-TM patients and controls.

*MTHFR C677T*	β-TM patients (n = 66) (%)	Controls (n = 66) (%)	OR	95% CI	P- value
CC	48 (73%)	60 (89%)	1	Ref	
CT	10 (15%)	4 (8%)	2.4	0.7–7.6	0.1
TT	8 (12%)	2 (3%)	4.9	1.2–24.2	0.03
C allele	106 (80%)	124 (93%)	1	Ref	
T allele	26 (20%)	8 (7%)	3.3	1.5–7.4	0.003

OR: odd ratio, CI: confidence interval, ref: reference.

### Biochemical Measurements of Patients with β-TM According to Their *MTHFR C677T* genotypes

**Fig 2:**
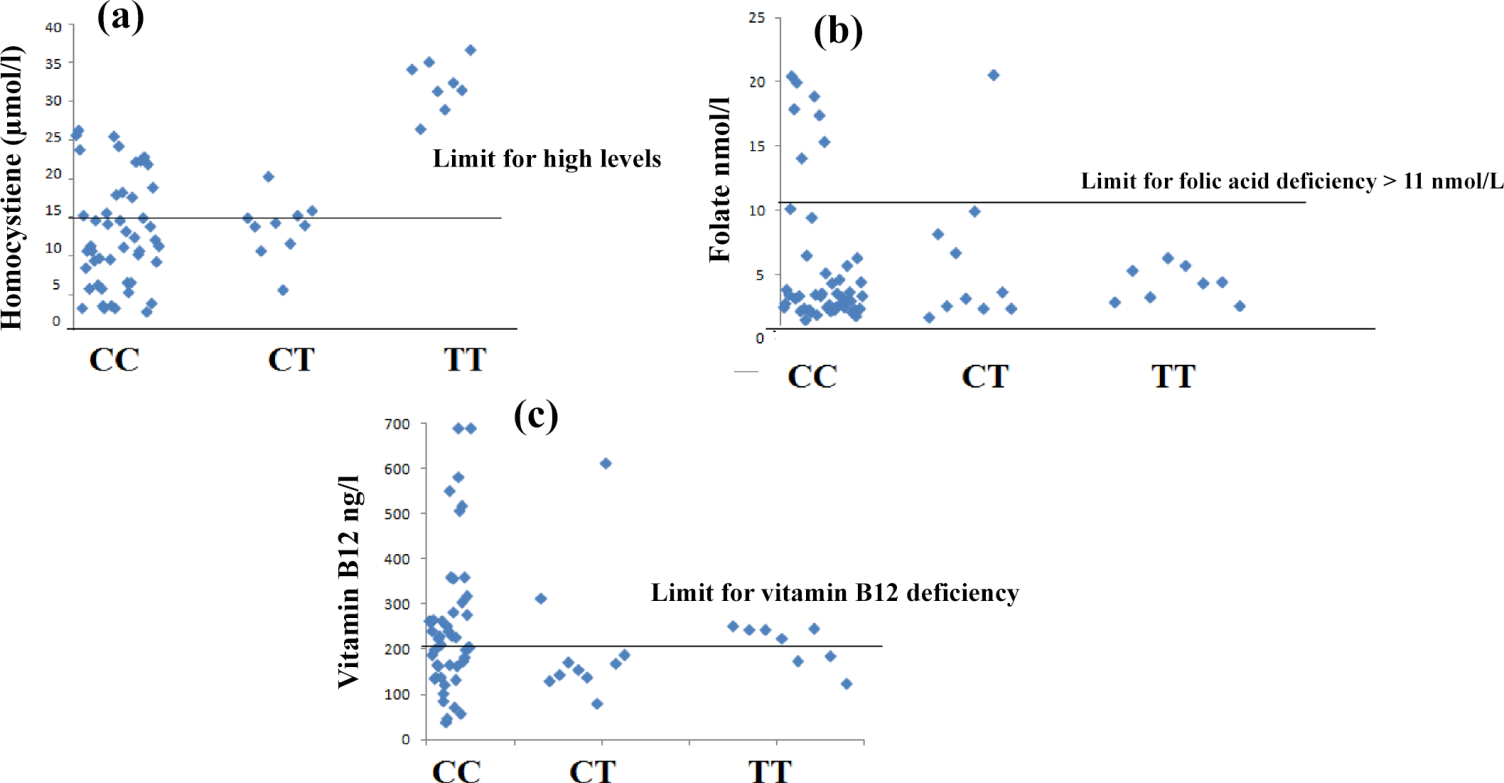
Scatter plots of plasma homocysteine A), folate (B) and vitamin B12 (C) concentrations in β-TM patients according to their *MTHFR* genotypes.

Regarding, the prevalence of Hhcy in patients with β-TM according to their *MTHFR* genotypes, the data show a strong and clear association between Hhcy and possession of *MTHFR TT* genotype where all patients with the *TT* genotype (100%) were assessed hyperhomocystinemic (≥ 15 μmol/l) compared with those with *CT* (40%) or the *CC* genotypes (29%) at P<0.001 (Fig 2A). Importantly, the frequency of the mutant *T* allele in patients with β-TM was strongly associated with Hhcy with an estimated OR of 7.7 (95% CI: 2.8–20.9), whereas no significant association was noticed with *CT* genotype indicating an increasing trend to the incidence of Hhcy with *TT* genotype in patients with β-TM (Table 4).

**Table 4:** Prevalence of hyperhomocysteinemia in β-TM patients according to their *MTHFR* genotypes.

*MTHFR* C677T	Homocysteine levels, n (%)				
	< 15 μmol/l	≥ 15 μmol/l	OR	95% CI	P- value
**CC**	34 (71%)	14 (29%)	1	Ref	
**CT**	6 (60%)	4 (40%)	1.6	0.3–6.6	0.7
**TT**	0	8 (100%)	∞	∞	P<0.001
**C allele**	74 (70%)	32 (30%)	1	Ref	
**T allele**	6 (24%)	20 (76%)	7.7	2.8–20.9	P<0.01

OR: odd ratio, CI: confidence interval, ref: reference

**Fig 3:**
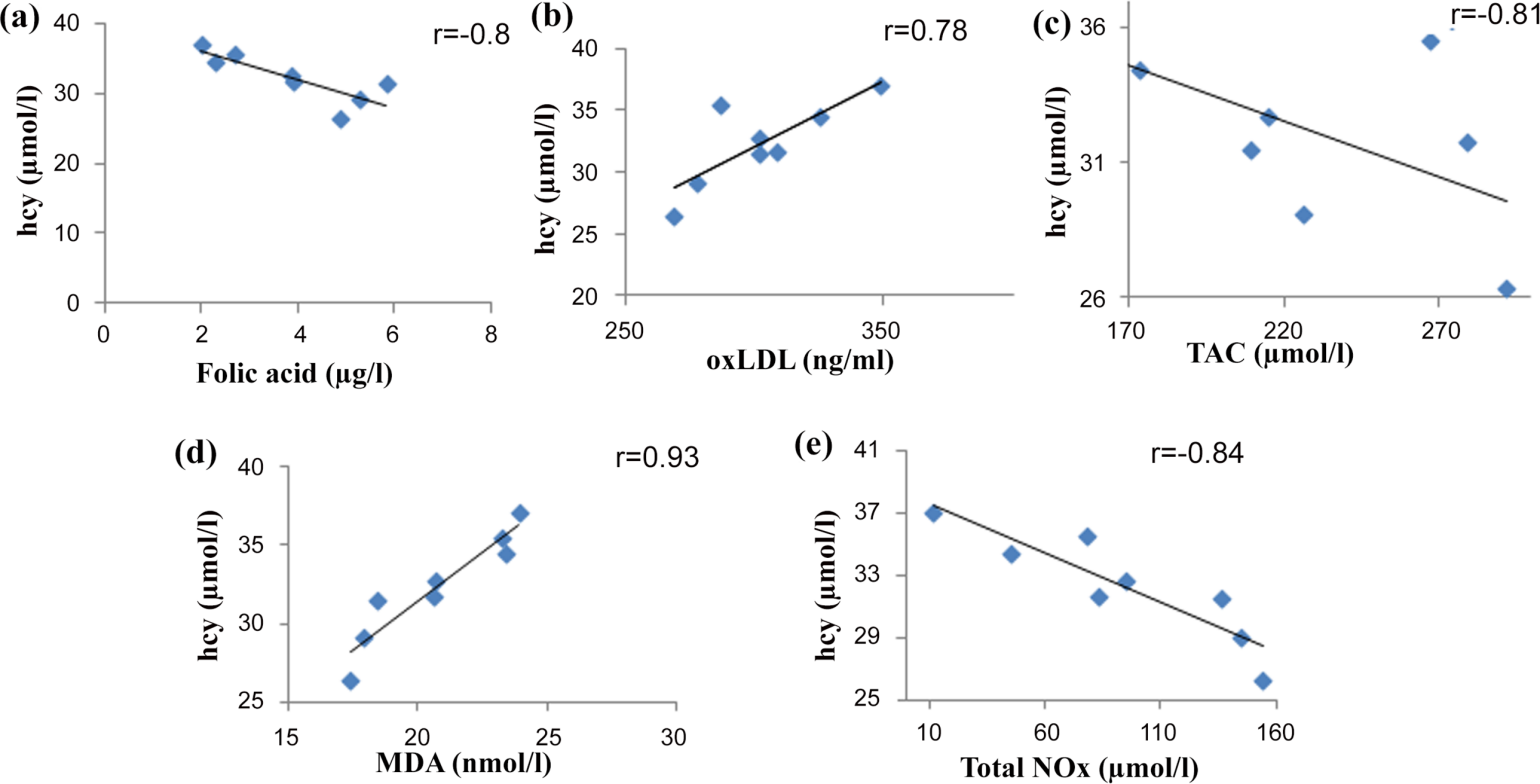
**Pearson correlation between hcy and oxLDL (A), TAC (B), MDA (C) and total NOx (D) in MTHFR 677TT genotype in β-TM patients.** hcy homocycteine, oxLDL oxidized low density lipoprotein, MDA malondialdehyde, TAC total antioxidant capacity, total NOx total nitric oxide.

Plasma vitamin B12 and folate levels were not significantly different between *MTHFR* genotypes among patients with β-TM. However, all Hhcy patients with *TT* had sub-optimal folate level and their plasma hcy was negatively correlated with plasma folate (r = -0.8, p = 0.01) (Table 5, Figs 2B and 3A).

**Table 5:** Biochemical parameters of β-TM patients according to their *MTHFR* genotypes.

***MTHFR* C677T Genotypes**			
	CC (n = 48)	CT (n = 10)	TT (n = 8)
**hcy (**μ**mol/l)**	12.80 ± 0.90	13.90 ± 1.20	32.30 ± 1.13 ^ab^
**Vitamin B12 (ng/l)**	248 ± 24	172 ± 18	227 ±16
**Folic acid (**μ**g/l)**	5.60 ± 0.76	6.10 ± 1.90	3.86 ± 0.50
**HDL-C (mg/dl)**	46.40 ± 2.09	34.8 ± 2.90	29.50 ± 2.80 ^a^
**LDL-C (mg/dl)**	122.70 ± 9.02	120.40 ± 16.10	145 ± 23.20
**AIP**	0.39 ± 0.02	0.54 ± 0.05	0.57 ± 0.01^ab^
**HDL/LDL**	0.54 ± 0.08	0.35 ± 0.07	0.26 ± 0.06 ^a^
**oxLDL (ng/ml)**	189 ± 14.70	292 ± 54.10^a^	302.60 ± 9.10 ^a^
**MDA (nmol/ml)**	15.40 ± 0.58	16.40 ± 1.54	20.68 ± 0.92^a^
**TAC (**μ**mol/l)**	303.50 ± 10.30	297.70 ± 16.6	212.60 ± 16.40^ab^
**Total NOx (**μ**mol/l)**	145.90 ± 9.50	116.30 ± 27	81.10 ± 15.80 ^a^

Data are represented as mean ± SE. Significant difference (a) from *CC* at P< 0.05 and (b) from *CT* at P< 0.05

AIP Atherogenic index of plasma, hcy homocysteine, HDL-C high density lipoprotein, LDL-C low density lipoprotein, MDA malondialdehyde, oxLDL oxidized low density lipoprotein, TAC total antioxidant capacity, TC total cholesterol, TG triglycerides, total NOx total nitric oxide.

Among β-TM patients with *MTHFR 677TT* genotype, significant reduction in HDL-C and HDL/LDL ratio were observed as compared to those with *CC* genotype. The AIP was significantly increased in *TT* genotype compared to *CC* and *CT* genotypes in patients with β-TM, but LDL-C was not statistically different (Table 5).

Regarding the changes in oxidative stress markers in our patients according to their *MTHFR* genotypes, plasma oxLDL was found significantly higher in *TT* and *CT* genotypes than in *CC* genotype. Plasma MDA was significantly increased in *TT* genotype compared to *CC* genotype. While, plasma TAC was significantly decreased in *TT* genotype compared to *CC* and *CT* genotypes and plasma total NOx was significantly decreased only in *TT* genotype compared to *CC* genotype (Table 4). In patients with *TT* genotype, plasma hcy was positively correlated with both plasma oxLDL (r = 0.78, P = 0.02) and plasma MDA (r = 0.93, P<0.01). Whereas, plasma hcy was negatively correlated with both TAC (r = -0.81, p = 0.01) and total NOx (r = -0.84, p<0.01) (Fig 3).

## Discussion

The current study was conducted to evaluate the prevalence of *C677T* polymorphism in *MTHFR* gene that encodes enzyme involved in folate-and vitamin B12-dependent hcy metabolism and its prospective contribution to Hhcy and related oxidative stress changes and plasma lipid indices modulations in Egyptian patients with β-TM.

The majority of cases of Hhcy are thought to be caused by interplay between micronutrients and genetic factors regulating one-carbon metabolism. Nutritional adequacy of B vitamins including folate and vitamin B12 is essential to maintain the plasma hcy within a normal homeostatic range [6]. In this study, plasma hcy concentrations were found significantly higher in patients with β-TM together with significantly lower plasma folate and vitamin B12 levels as compared with those in control subjects. Such, decreases in the plasma level of both micronutrients in patients with β-TM might be attributed to inadequate intake in the face of increased consumption and demand in patients with β-TM [26]. Importantly, the increment in serum ferritin often observed in patients with β-TM can catabolize folate *in vitro* and *in vivo* causing a reduction in its serum level [13]. These results about Hhcy along with B vitamins deficiency in our patients; are in accordance with several studies conducted on other clinical conditions, which demonstrated that deficiency of B vitamins can lead to elevated plasma hcy level [27].

With regards to the genetic factor predisposing to Hhcy, the *C677T* thermolabile variant of the *MTHFR* gene is an important genetic determinant of hcy metabolism. In this study, individuals homozygotes for the mutant *MTHFR 677TT* genotype were more frequently recorded in patients with β-TM (12%) than in the control group (3%). Additionally, the frequency of *T* allele was found significantly higher in patients with β-TM than in controls revealing a significant association between β-TM and *MTHFR C677T* gene polymorphism. This is the first documentation revealing a significant association between this polymorphism and patients with β-TM. In similar study, Zalloua et al. [28] reported high prevalence of *MTHFR C677T* gene polymorphism on Lebanese patients with β-thalassemia intermedia. These data are in agreement with a higher prevalence of the homozygous mutant genotype *TT* in Mediterranean countries compared to other countries in Northern Europe [29]. On the contrary, earlier studies in Iran by Rahimi et al. [30] and in Kuwait by Mustafa et al. [31] failed to find any association between β-TM and such a mutation. However, in another Hb disorder, Neto et al. [32] reported increased frequency of *MTHFR C677T* polymorphism among Brazilian patients with sickle cell anemia.

Concerning the prevalence of Hhcy amosng the different *MTHFR* genotypes in our patients, all patients with *TT* genotype (100%) were assessed as hyperhomocystienemic (≥15 μmol/L) relative to those with the *CT* (40%) or the *CC* genotype (29%). These data show a clear and a strong association between Hhcy and the frequency of the mutant *T* allele indicating an increasing trend in the development of Hhcy among our patients with *TT* genotype. These observations confirm the results of preceding studies conducted on atherosclerotic patients [33].

Moreover, our results showed that although plasma folate and vitamin B12 concentrations were not significantly different among the three genotypes, a sub-optimal folate level was observed only in patients who were homozygotes for the *MTHFR* along with higher fasting hcy level. Meanwhile, their plasma hcy was negatively correlated with plasma folate. This is consistent with early reports of Papoutsakis et al. [7] and Bailey and Gregory III [34] confirming an association between the *MTHFR TT* genotype and low serum folate in increasing the risk of development of Hhcy. An explanation for these observations could be based on hypothesis that a higher folate status may increase the *in vivo* stability of the *MTHFR* enzyme, thus reducing the difference in enzyme activity between the *TT* and *CT* or *CC* genotypes [35]. Another explanation for this could be that folate protected this enzyme against flavin loss and inactivation as suggested by Guenther et al. [36]. Accordingly, subjects with *MTHFR TT* genotype could have an exaggerated Hhcy due to the decrement of folic acid [37]. In β-TM patients, chronic ongoing hemolysis causes a high demand for folate, necessary for normal erythropoiesis [12] which may aggravate the detrimental effect of *MTHFR 677 TT* induced -Hhcy on the vascular system.

Regarding lipid profile in patients with β-TM, TC and TG were not significantly varied with respect to control values, however their cholesterol-carrying lipoproteins were markedly perturbed. Thus, HDL-C and HDL/LDL were markedly lowered, while LDL-C and AIP were markedly elevated in our patients compared to the controls. These data pointed out that patients with β-TM are likely to be at increasing risk for development of atherogensis. These unfavorable changes in lipid profile were more adversely affected in patients with the *TT* genotype than in those with *CC* genotype where their HDL-C and HDL/LDL were significantly lower than in *CC* genotype group. This could be explained based on Hhcy observed in our patients particularly, those with *TT* genotype. Hhcy has been stated to reduce the concentration of plasma HDL-C by inhibiting the hepatic synthesis of apoA-I, the main HDL apolipoprotien [38,39]. HDL has several properties that may contribute to their antiatherogenic potential especially apoA-I has been shown to have antioxidant properties. This mechanism may link Hhcy to the development of atherosclerosis as had been previously reported [40]. Furthermore, AIP the most important prognostic marker for vascular disease, was more significantly higher in patients with *TT* genotype than in *CC* or *CT* genotypes, suggesting that *TT* patients with Hhcy are more vulnerable to a higher risk for development of atherosclerosis as explored by other investigators in other pathologic conditions [41,42]. Importantly, AIP can be used as an index for the risk assessment of atherosclerosis even when the other lipid parameters are not correlated with the disease.

In the current study, patients with β-TM were found in state of oxidative stress indicated by marked increment in plasma MDA and oxLDL together with a remarkable decrement in plasma TAC as compared with the healthy control. Earlier, it has been demonstrated that there was increased and uncompensated oxidative stress in β-thalassemia intermedia [43] and β-TM [12] patients. In fact, oxidative stress is a consequence of the disease process in β-TM where chronic hemolysis and repeated blood transfusion with accumulation of hemolytic cell products tremendously increased body iron burden in these patients [44]. Under these conditions, increased free radicals production, enhanced peroxidative damage to tissues components and depletion of endogenous antioxidants would be expected triggering the embarrassed oxidative stress changes among patients with β-TM in this study. Furthermore, the ongoing oxidative stress in β-TM is exacerbated in patients with *TT* genotype, where their plasma levels of oxLDL and MDA, key elements of atherogensis were substantially increased and their plasma TAC concentration was severely decreased as compared with those in *CC* genotype. On analyzing the relationship between plasma oxidative stress markers and plasma hcy in patients with *TT* genotype, a strong positive correlation between plasma hcy and both MDA and ox.LDL as well as a high negative correlation between plasma hcy and TAC were obtained in those patients. These data underscore the potential role of Hhcy in mediating the prooxidative conditions in patients with *TT* genotypes as previously reported in other studies [45–47]. Hhcy mediated cytotoxicity is, in part, attributable to the generation of reactive oxygen species *via* autooxidation of the SH groups in hcy to homocystine or other mixed disulfides, which would be enhanced by iron burden in those patients [8].

In our study, the levels of nitrate/nitrite (NOx), metabolites of NO were significantly lower in patients with β-TM as compared with control group. Factors that influence NO bioavailability are likely to be of considerable clinical importance. NO is an antiatherogenic molecule and its bioavailability has been demonstrated to be decreased in conditions including increased hemolysis, inflammation and oxidative stress [48], such conditions are similar to that occurred in patients with β-TM. Previously, Reiter et al. [49] showed that free Hb caused vascular dysfunction by disturbing NO bioavailability in chronic hemolytic diseases. Regarding the impact of *MTHFR* gene mutation on NOx levels, patients with *TT* genotype had severely decreased plasma NOx compared to those with *CC* genotype. Moreover, a strong negative correlation between plasma hcy and NOx has been observed among those patients inconsistent with preceding studies [50,51] in other clinical disorders. Hhcy has been reported to impair the synthesis of NO by inhibiting the activity of dimethylarginine-dimethylamino-hydrolase activity and increasing the accumulation of asymmetric dimethylarginine, thus reducing NO production [52]. In addition to that, long-term exposure to hcy has been demonstrated to impair endothelial production of NO [9]. This could be explained based on excessive formation of peroxynitrite (formed by the reaction of NO with superoxide anion) during Hhcy. Peroxinitrite may activate poly (ADP-ribose) L-polymerase which is an important mediator of vascular dysfunction [53]. The decreased levels of HDL may have a role in the reduction of NO bioavailability, where HDL correct endothelial dysfunction by inducing the synthesis of nitric oxide and promoting endothelial repair [40]. Accordingly, low NO values in patients with β-TM may contribute to the development of atherogenic complications particularly those due to endothelial dysfunctions.

## Conclusion

The presented and described findings of this work provide a strong evidence for the interaction between the *C677T MTHFR* polymorphism and plasma B-vitamins particularly low folate on the development of Hhcy in Egyptian patients with β-TM. The present data underscore the potential role of Hhcy in mediating the oxidative changes and other related vascular complications in β-TM patients indicated by enhanced lipid peroxidation, increased oxLDL and atherogenic lipid indices as well as reduction of TAC and NO bioavailability. Therefore, patients with β-TM should be monitored routinely for their plasma hcy and oxidative stress markers to alleviate the severity of anemia and to introduce preventive strategies early and before situations of atherogenicity have developed.

## Supporting Information

S1 Table The demographic characteristics of the studied groups. (DOCX)

S2 Table Pearson correlation between homocysteine and other biochemical parameters in *β*-TM patients with *MTHFR 677TT* genotype. (DOCX)

S3 Table Pearson correlation between oxLDL and other biochemical parameters in *β*-TM patients with MTHFR 677*TT* genotype. (DOCX)

## Abbreviations

AIP: Atherogenic index of plasma
ALT: Alanine transaminase
ALP: Alkaline phosphatase
AST: Aspartate transaminase
BSA: Body surface area
β-TM: β-thalassemia major
Hb: Hemoglobin
Hcy: Homocystiene
HDL-C: High density lipoprotein-cholesterol
Hhcy: Hyperhomocystienemia
LDL-C: Low density lipoprotein-cholesterol
MDA: Malondialdehyde
MTHFR: Methylenetetrahydrofolate reductase
OxLDL: Oxidized low density lipoprotein
PCR-RFLP: Polymerase chain reaction-Restriction fragment length polymorphism
PCV: Packed cell volume
RBCs: Red blood cells
TAC: Total antioxidant capacity
TC: Total cholesterol
TG: Triglycerides
Total NOx: Total nitric oxide
WBCs: White blood cells
