# Supplementary material for: Impact of Genetic Polymorphism of methylenetetrahydrofolate reductase C677T on Development of Hyperhomocysteinemia and Related Oxidative Changes in Egyptian β-Thalassemia Major Patients
Source: PLoS One. 2016 May 17;11(5):e0155070. doi: 10.1371/journal.pone.0155070 (PMC4871363; doi:10.1371/journal.pone.0155070)
Supplement: S1 Table — (DOCX) [file pone.0155070.s001.docx]

| **Groups**  **Parameters** |  | ***β*-TM** **patients**  **(n=66)** | **Control subjects**  **(n=66)** | **P value** |
| --- | --- | --- | --- | --- |
| **Age (years)**  **Min-Max** |  | 15.4 ± 0.69  (8 - 26) | 16.3 ± 1.2  (9 - 28) | 0.431 |
| **Sex** | **M** | 30 (45%) | 34 (56%) |  |
|  | **F** | 36 (55%) | 32 (44%) |  |
| **Weight (kg)** |  | 39.2 ± 1.4  (23 - 60) | 46.6 ± 1.3  (28 - 65) | 0.821 |
| **Height (cm)** |  | 140.3 ± 1.9  (95 - 170) | 145.2 ± 2.8  (120-165) | 0.943 |
| **BSA (m^2^)** |  | 1.22 ± 0.03  (0.6 - 1.7) | 1.36 ± 0.04  (0.97 - 1.72) | 0.853 |
| **Age of diagnosis**  **(month)** |  | 21.2 ± 2.06  (4 - 60) | - |  |
| **Volume of blood transfused per year (L)** |  | 4.3 ± 0.15  (0.6 - 7.2) | - |  |
| **Family history** | **Yes** | 11 (17%) | - |  |
|  | **No** | 55 (83%) | - |  |
| **Splenectomized** | **Yes** | 42 (64%) | - |  |
|  | **No** | 24 (36%) | - |  |
| **Hepatomegaly** | **Yes** | 20 (30%) | - |  |
|  | **No** | 46 (70%) | - |  |
| **Mongoloid features** | **Yes** | 30 (45%) | - |  |
|  | **No** | 36 (55%) | - |  |

**S1Table. The demographic characteristics of the studied groups.**

Data are represented as mean ± SE. Range or percentage is given in parenthesis.

P value ˃ 0.05 is non significant. BSA body surface area, *β*-TM *β* thalassemia major
