# Supplementary material for: Impact of Genetic Polymorphism of methylenetetrahydrofolate reductase C677T on Development of Hyperhomocysteinemia and Related Oxidative Changes in Egyptian β-Thalassemia Major Patients
Source: PLoS One. 2016 May 17;11(5):e0155070. doi: 10.1371/journal.pone.0155070 (PMC4871363; doi:10.1371/journal.pone.0155070)
Supplement: S2 Table — (DOCX) [file pone.0155070.s002.docx]

**S2 Table. Pearson correlation between homocystiene and other biochemical parameters in *β*-TM patients with *MTHFR 677TT* genotype.**

| **Parameters** | **r** | **Correlation** | **P value** | **Significance** |
| --- | --- | --- | --- | --- |
| **Vitamin B12** | -0.27 | Negative | 0.517 | Not significant |
| **Folate** | -0.81 | Negative | 0.014 | Highly significant |
| **oxLDL** | 0.78 | Positive | 0.022 | Significant |
| **TAC** | -0.81 | Negative | 0.014 | Highly significant |
| **MDA** | 0.93 | Positive | 0.0008 | Highly significant |
| **Total NOx** | -0.84 | Negative | 0.008 | Highly significant |

P<0.05 is considered significant, P< 0.01 is considered highly significant.

MDA malondialdehyde, , oxLDL oxidized low-density lipoprotein, TAC total antioxidant capacity
