# Supplementary material for: Impact of Genetic Polymorphism of methylenetetrahydrofolate reductase C677T on Development of Hyperhomocysteinemia and Related Oxidative Changes in Egyptian β-Thalassemia Major Patients
Source: PLoS One. 2016 May 17;11(5):e0155070. doi: 10.1371/journal.pone.0155070 (PMC4871363; doi:10.1371/journal.pone.0155070)
Supplement: S3 Table — (DOCX) [file pone.0155070.s003.docx]

**S3 Table. Pearson correlation between oxLDL and other biochemical parameters in *β*-TM patients with MTHFR 677*TT* genotype**

| **Parameters** | **r** | **Correlation** | **P value** | **Significance** |
| --- | --- | --- | --- | --- |
| **Vitamin B12** | -0.51 | Negative | 0.196 | Not significant |
| **Folate** | -0.68 | Negative | 0.0635 | Not significant |
| **TAC** | -0.71 | Negative | 0.048 | Significant |
| **MDA** | 0.74 | Positive | 0.035 | Significant |
| **Total NOx** | -0.91 | Negative | 0.001 | Highly Significant |

P<0.05 is considered significant, P< 0.01 is considered highly significant.

MDA malondialdehyde, oxLDL oxidized low density lipoprotein, TAC total antioxidant capacity
